# Immunosuppressive arenaviral exoribonuclease

**DOI:** 10.18632/oncotarget.6110

**Published:** 2015-10-13

**Authors:** Bjoern Meyer, Hinh Ly

**Affiliations:** Department of Veterinary and Biomedical Sciences, University of Minnesota, Twin Cities, MN, USA

**Keywords:** arenavirus, immune evasion, Lassa virus, nucleoprotein, exoribonuclease, Immunology and Microbiology Section, Immune response, Immunity

Arenaviruses cause up to 500,000 zoonotic infections per year in endemic areas of Africa and South America and can lead to severe and lethal hemorrhagic fever (HF) symptoms [1]. Currently, there is no specific antiviral drug or vaccine available for the treatment of these infections, with the exception of the Candid #1 vaccine that has successfully been used to prevent infections of Junin virus in the endemic areas of Argentina. Progression and severity of arenaviral HF disease generally correlates with viremia and host immune suppression, the molecular mechanisms of which have only recently been revealed [2]. Mounting an early and effective induction of the host innate-immune response to the infection, which is followed by robust cell-mediated responses, is thought to be essential to control the outcome of the infection. Many arenaviruses, especially those belonging to the Old World virus group, have developed different strategies to effectively suppress the host immune responses to infections.

Our laboratory has been involved in characterizing the molecular mechanisms behind innate immune suppression mediated by arenaviral nucleoprotein (NP). Our high-resolution structure of the Lassa virus (LASV) NP reveals a conserved DEDDh exonuclease in its C terminal domain [3], which can degrade double-stranded RNA that would normally trigger the type I interferons (IFN) signaling pathway upon viral infections. NP has also been shown to interfere with the proper functions of several factors in the RIG-I-like receptor (RLR) pathway, namely IKKε to prevent IRF3 activation, and the Nuclear Factor Kappa B (NF-κB) pathway, and therefore suppressing the production of IFN-β and potentially other cytokines [4]. Initial studies using NPs expressed from plasmid DNAs have attributed the non-pathogenic nature of the Tacaribe virus (TCRV) to a change of amino acids around the DEDDh exonuclease site and the observed lack of IRF3 inhibition. Our efforts to resequence TCRV and to perform a comprehensive structure-function analysis of the TCRV NP have revealed discrepancies with the GenBank deposited sequence and shown that the IFN-antagonism displayed by the NP exoribonuclease (RNase) activity is a conserved mechanism among different arenaviruses, including but not necessarily limited to TCRV, LASV and Pichinde virus (PICV) [5]. Using recombinant viruses, we have recently shown the importance of the IFN-suppressive activity of NP and its role in mediating optimal viral replication in cell culture as well as in a surrogate animal model of LASV infection that is based on PICV-infected guinea pigs [5]. Specifically, we show that mutating each of the NP's catalytic DEDDh residues to alanines results in significantly reduced levels of IFN-β inhibition and virus grow potentials in immune-competent cells, and in an attenuated phenotype of the mutant viruses in infected animals. The important role of the NP RNase function in suppressing immune response in order to favor virus replication is evidenced by the appearance of wild-type virus revertants in many of the sick animals with detectable levels of viremia [5].

Our findings are supported by recent studies with recombinant LASV carrying the NP catalytic double mutations (D389T/G392A and D389A/G392A), which lose the ability to suppress the type I IFN-induction pathway in dendritic cells or macrophages, which are thought to be primary target cells for virus infection [6, 7]. The inability of the LASV NP catalytic mutants to suppress type I IFNs results in upregulated expression levels of major histocompatibility complex (MHC) class I gene pathways as well as important macrophage-derived cytokines that activate natural killer (NK) cells to participate in the direct killing of the infected cells. Thus, the proper function of the NP RNase appears to be essential in suppressing the early innate immune response, which directly correlates with the priming of cellular immunity and therefore impacts virus clearance *in vivo*.

Taken together, studies conducted in our laboratory as well as by other laboratories have provided strong evidence to support the role of the arenaviral NP exoribonuclease function in mediating immune suppression upon viral infections. It is important to highlight the fact that this is the first time that a viral exoribonuclease function has been attributed to mediating host immune evasion. Because there is currently no specific antiviral therapy available against arenaviruses, a better level of understanding of how arenaviruses are able to evade host immunity is warranted as it may offer new ways to evaluate future treatment options and to design and test vaccine candidates against these deadly human viral pathogens.
